# Perspective on the Application of Machine Learning Algorithms for Flow Parameter Estimation in Recycled Concrete Aggregate

**DOI:** 10.3390/ma16041500

**Published:** 2023-02-10

**Authors:** Justyna Dzięcioł, Wojciech Sas

**Affiliations:** 1 Institute of Civil Engineering, Warsaw University of Life Sciences, 159 Nowoursynowska, 02-776 Warsaw, Poland; 2 Water Centre, Warsaw University of Life Sciences, 159 Nowoursynowska, 02-776 Warsaw, Poland

**Keywords:** permeability coefficient, recycled aggregates, prediction, machine learning, k-Nearest Neighbors, artificial neural network, SHAP

## Abstract

The constantly expanding civilization and construction industry pose new challenges for a sustainable development economy. Aiming to protect the environment is often associated with waste management, thereby reducing the number of landfills. The management of recycled concrete aggregate (RCA) from building demolition and its reuse in construction perfectly fits into this trend. The characteristics of post-industrial and recycled materials are not homogeneous as is usually the case with natural materials. This leads to a search for solutions to determine the parameters in the simplest possible manner and with as few resources as possible, while eliminating estimation risks. This task can be solved using machine learning, whose algorithms are increasingly used and developed in many areas of life and industry. The research in this study is aimed at comparing the effectiveness of k-Nearest Neighbors (k-NN) and Artificial Neural Network (ANN) algorithms in determining the permeability coefficient to a linear regression model. This parameter has an important role from the perspective of the application of RCA in civil engineering, particularly in earth construction. Two different RCA materials with different origins and properties were used in the study. The filtration test for each sample was pre-prepared using different compaction energies of 0.17 and 0.59 J/cm^3^ and for loosely packed samples. Differences in the structures of the test results are presented for both materials. The lowest prediction errors were obtained for the k-NN model. This algorithm obtained for the training sample a coefficient of determination (R^2^) equal to 0.947 and for the test sample an R^2^ equal to 0.980. In the case of ANN, the coefficient of determination was in the range of 0.877–0.936. An important part of the study was the interpretation with SHAP of the obtained models, allowing insight into which parameters influenced the predictions. That is significant and novel, considering the heterogeneity of the materials studied, and provides a rationale for further research in this area.

## 1. Introduction

The construction industry is one of the largest and most active industrial sectors in the world, using more raw materials and energy than any other economic activity. As a result, the waste generated by this activity accounts for a significant portion of the total waste generated [1,2]. To curtail the disposal of demolition waste, concrete aggregate is crushed and reused as a substitute for natural aggregates [3]. Recycled aggregates used in earth structures are usually cheaper than natural aggregates, which is important when deciding on the construction process. Reusing construction waste is also an environmentally sustainable solution [2,4,5,6,7,8].

Recycled construction aggregate frequently represents waste generated during construction and demolition (C&D) and arises throughout the life cycle of buildings, including during the planning and design phases [1]. In recent decades, it has gained attention, and an economic model—circular economy—has its foundations in better management of resources [9], whose goal is to keep materials in a closed circle at their highest value [1,10].

The properties of anthropogenic aggregates differ from those of natural aggregates and are related, among other things, to the considerable diversity in the composition of these materials [11,12]. This influences the constant search for solutions related to supporting the determination of parameters of anthropogenic aggregates. However, one parameter of particular importance in the planning of earth structures is the coefficient of permeability, which depends on the grain size, structure, and porosity of the material. Filtration properties affect foundation conditions in the design and implementation of geotechnical structures. Previously, the coefficient of permeability was determined mainly by statistical linear and non-linear prediction methods and based on the principles of Darcy’s law [13,14,15].

The original purpose of machine learning was to automate information extraction processes that would allow quicker and simpler access to expert knowledge [16]. Practical applications of machine learning techniques and algorithms differ significantly from empirical or theoretical studies, involving aspects of data collection and organization, as well as the selection of adequate algorithms [17,18]. Over the years, many algorithms have been developed to solve various complicated research problems of both regression and classification [16,19,20]. Machine learning methods have been extensively used in recent decades to generate predictive models of material properties [18,21,22]. Support vector regression (SVR) was used by Mishra et al., (2021) to predict the probabilistic design of a retaining wall [23], and Amin et al., (2021) used an artificial neural network (ANN) to design the optimal portions content of RHAC [24]. Lechowicz and Sulewska (2023) applied ANN to develop empirical relationships used in a preliminary design to evaluate the settlement and unconfined shear strength of embankment-loaded organic soil [25], while Chou et al., (2016), among others, used data mining including linear regression, classification, and regression tree analysis (CART) to identify factors affecting the shear strength and predict the peak FRS friction angle [26]. This could have generated a new problem of selecting appropriate algorithms for solving specific engineering problems. However, the development and availability of machine learning techniques provided an opportunity to test their usefulness in determining the parameter of the coefficient of permeability.

The solution to predictive problems began with linear regression based on the method of least squares, which was introduced by Legendre in 1805 [27]. The rapid expansion of computer performance has gradually become an important factor propelling the development of artificial intelligence (AI). The concept of AI debuted at the Dartmouth Conference in 1955, presented by several computational scientists including John McCarthy, Marvin Minsky, and Claude Shannon. Their original idea was to use machines to imitate human learning [28].

The perceptron, which was invented in 1958, by Professor Frank Rosenblatt [29], was used to develop artificial neural networks, which are a popular machine learning technique for analyzing data through a network of decision layers. A typical neural network consists of an input layer, a hidden layer, and an output layer. The data are first received by the input layer, where features are detected. The hidden layer or layers then analyze and process the input features, and the final result is presented as an output layer [30,31,32,33,34,35].

The k-Nearest Neighbors (k-NN) algorithm is a type of supervised machine learning algorithm. k-NN is extremely easy to implement in its most basic form, yet it performs quite complex classification tasks. k-NN is a non-parametric learning algorithm, which means that it assumes nothing about the underlying data. This is extremely useful because most real-world data do not meet theoretical assumptions, e.g., linear separation and uniform distribution [36,37,38,39].

Two recycled concrete aggregate materials of different origins and compositions were used for the research in this article. The authors aimed to verify the suitability of the k-NN and ANN algorithms for the prediction of an optimized and reliable model for determining the coefficient of permeability in recycled concrete aggregate, taking into account the risks associated with the estimated model. The interaction of the properties of the two materials was analyzed. An important and novel element of the work was the analysis of the interaction of the properties of the two materials in the context of their influence on the estimated models. For this purpose, SHAP has been used to provide better insight into the performance of individual models and the impact of individual parameters on the models.

The database was implemented into Orange software, where selected algorithms were applied. The R programming language was used for the calculations and the presentation of the analysis results.

## 2. Materials and Methods

### 2.1. Materials and Filtration Study Method

The constant head method was used to test the permeability characteristics for RCA. The method is characterized by simplicity and unchanging test conditions, and the constant head method alone is one of the most reliable techniques for measuring permeability in non-cohesive soil [15].

**Figure 1:**
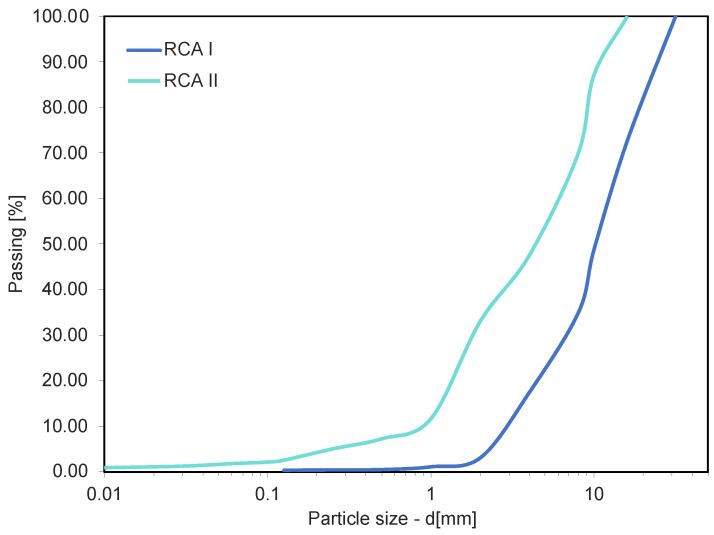
Grain size curve of tested materials.

Two types of recycled aggregate obtained from crushed demolition concrete were used in the permeability coefficient study. The materials originated from different demolitions. The grain size curves of the two materials are presented in Figure 1.

The parameters describing the aggregates that were taken for further analysis were compaction energy (0.00 (control), 0.17, and 0.59 [J/cm^3^]), grain size (particle sizes d5, d10, d20, d30, d60, and d90 [mm]), dry density, specific density, porosity, and void ratio. The average values of these parameters are presented in Table 1.

### 2.2. Data Preparation and Overall Methodology for the Applied Machine Learning Algorithms

The application of machine learning algorithms requires the consideration of many issues related not only to the appropriate preparation of the database but also to the selection of the sampling method, the mechanism for learning models and the form of their operation, the ability to handle missing values, noisy or numerical data, the computational complexity of the algorithm, or learner–user interaction [40,41,42,43]. The selection of algorithms for further analysis was motivated by a comparison of the well-known and widely used for predictive tasks artificial neural network algorithm with the equally versatile, newer, but also less recognized for activity in the field of engineering analysis k-Nearest Neighbors algorithm. The following sections present brief characteristics of the analyzed algorithms.

**Figure 2:**
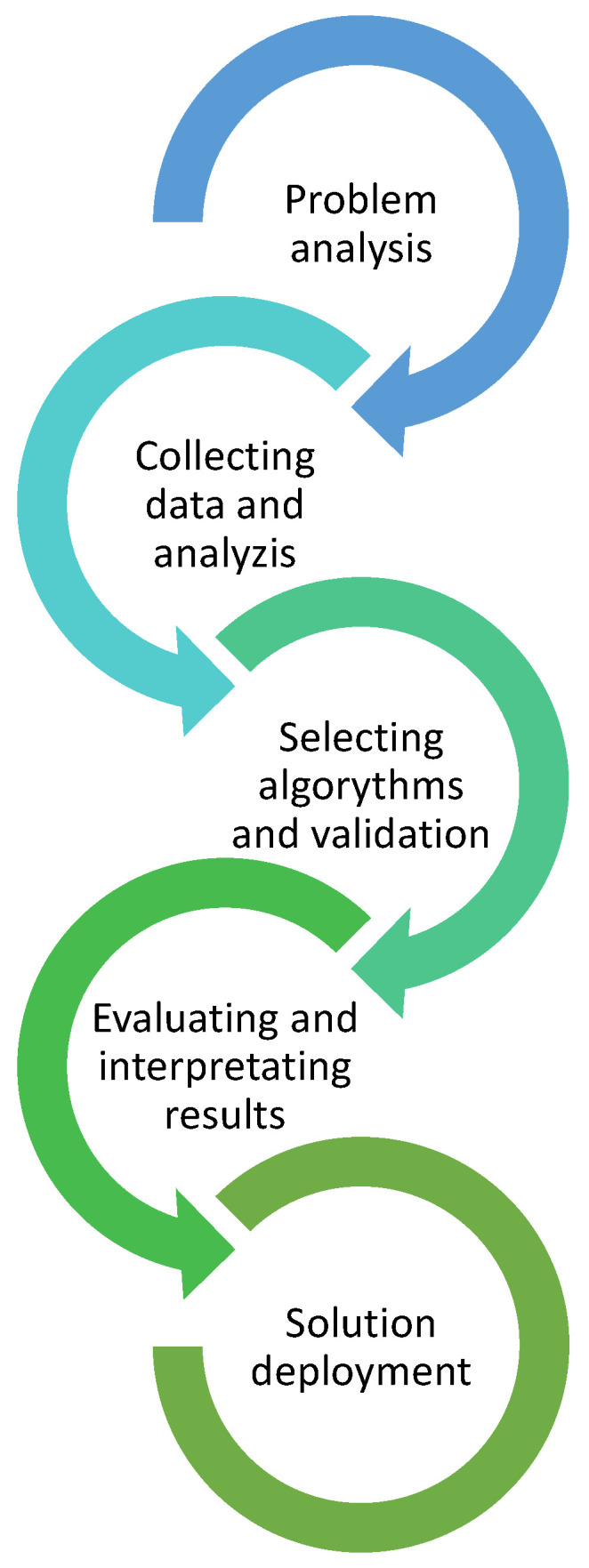
Analysis scheme using machine learning.

The first stage of the analysis was to collect the data, analyze them, and verify any erroneous or blank data for elimination or completion. This was one of the most important elements of the analysis as a reliable and well-prepared database was the basis for further calculations and inference. The general scheme of the procedure for modeling with machine learning algorithms is shown in Figure 2.

**Figure 3:**
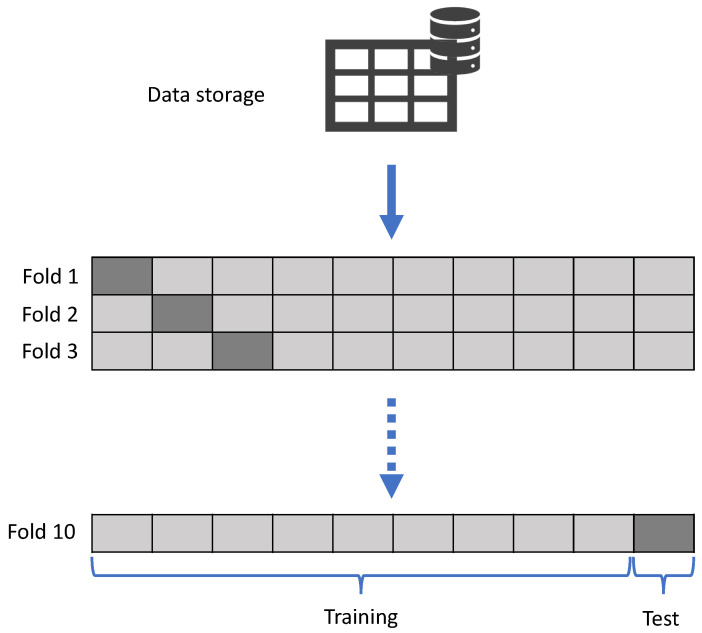
Estimation using the k-fold cross-validation method.

The k-fold cross validation technique (Figure 3) was chosen as the method for sampling and validating the model, a technique that minimized the drawbacks of the holdout. The k-fold cross validation produced more stable and reliable results since training and testing were performed on several different parts of the data set; in this case, 10 folds were included to test the model on many different subsets of the data [44,45].

### 2.3. Artificial Neural Networks

The Artificial Neural Networks algorithm is an information processing system formed by processing units named neurons. A neuron, which allows various inputs to be mapped to the output, is the most basic element in any Artificial Neural Network. They are a stack of computational units that result in a model with the required level of generic and overfitting error. When building a network within the implemented framework, assumptions were taken into consideration, such as topology, that is, the layout of connections between neurons. The training algorithm analyzed the obtained weights on the connections and selected the activation function.

### 2.4. k-Nearest Neighbors

k-Nearest Neighbors (k-NN) is an algorithm that is used in both classification and regression problems. In the analysis, it was used a variant of regression. The value of the k means, the calculated distances to find the neighbors for a given input, and proper matching of the number of classes affected the accuracy of the model. In the k-NN regression, the output was the average value of k-Nearest Neighbors.

Previous research did not use k-NN regression to determine the filter coefficient, and this algorithm has been weakly recognized in geoengineering solutions. The data preprocessing analysis was used to identify optimal k values. Through it, the number of k for the model was tuned to 2.

### 2.5. Error Analysis

The evaluation included the k-NN and neural network algorithm; linear regression was used as a reference and control algorithm. The results were verified with the use of error analysis, and the following values were estimated for individual models:

- Mean square error (MSE) represents the mean squared difference between the raw and predicted values in a data set. It measures the variance of the residuals.

(1) $$MSE=\frac{1}{N}\overset{N}{\underset{i=1}{\sum }}{\left(y_{i}-\hat{y}\right)}^{2}$$

- Root mean square error (RMSE) is the square root of the mean squared error. It measures the standard deviation of the residuals.

(2) $$RMSE=\sqrt{MSE}=\sqrt{\frac{1}{N}\overset{N}{\underset{i=1}{\sum }}{\left(y_{i}-\hat{y}\right)}^{2}}$$

- Mean absolute error (MAE) represents the average absolute difference between the observed and predicted values in a data set. It measures the average of the residuals in a data set.

(3) $$MAE=\frac{1}{N}\overset{N}{\underset{i=1}{\sum }}\left|y_{i}-\hat{y}\right|$$

- Coefficient of determination (R^2^) represents the portion of the variance of the dependent variable that is explained by the linear regression model. This is a scale-free result, i.e., whether the values are small or large, the R^2^ value will be less than one.

(4) $$R^{2}=\frac{\sum_{i=1}^{N}{\left({\hat{y}}_{i}-\bar{y}\right)}^{2}}{\sum_{i=1}^{N}{\left(y_{i}-\bar{y}\right)}^{2}}$$

## 3. Results and Discussion

**Figure 4:**
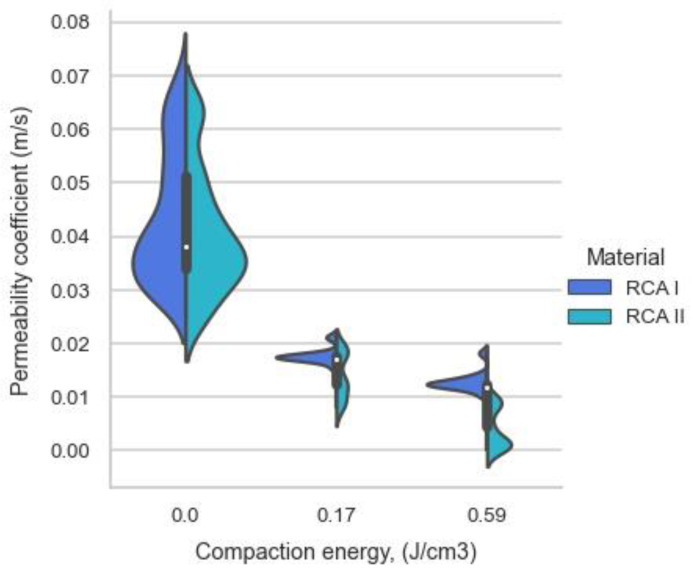
The relationship of the permeability coefficient results to compaction energy in the context of the tested material.

The material before testing was compacted at energies of 0.17 and 0.59, and some samples were tested without applying additional energy (control samples). The data collected from the tests were analyzed for their dependence on the applied compaction energy. The structure of the distribution of test results in relation to the applied compaction energy is presented in Figure 4. The scatter between the minimum and maximum values obtained from the tests was much larger when the material was compacted “freely” than in the cases of tests where the compaction energies of 0.17 and 0.59 J/cm^3^ were applied.

The characterization of the test results from the perspective of the given material allowed us to observe significant differences in the test results, which could be seen especially for specimens compacted with additional compaction energy. It should be added that the two materials tested retained different flow characteristics (coefficients of permeability) after compaction. However, the range of results obtained was similar. Factors such as the cement content, aggregate composition, or mineral composition could have influenced this [46,47,48].

**Figure 5:**
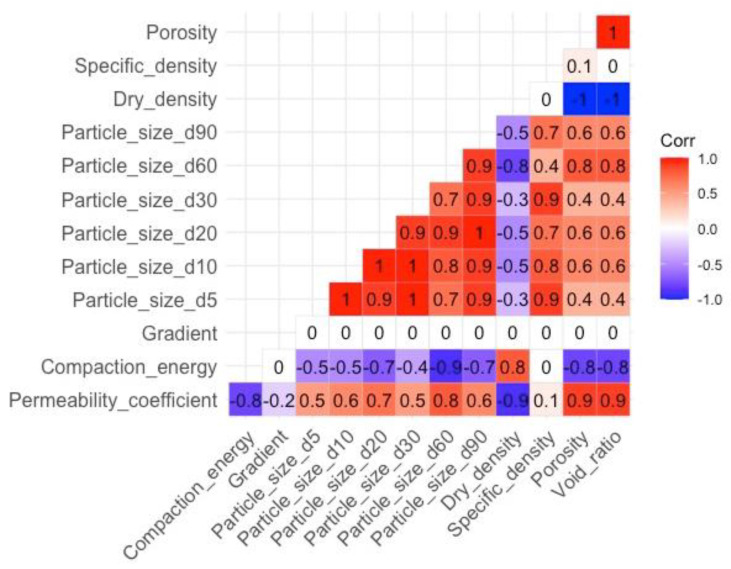
Correlation analyzed.

Figure 5 presents the correlations of the various properties with each other and the filtration coefficient. The heat map allows a preliminary assessment of the relevance and suitability of individual parameters for estimation purposes. The highest positive correlation with the permeability coefficient was obtained for porosity, void ratio (0.9), and particle size d60 (0.8), while at the opposite extreme were dry density (−0.9) and compaction energy (−0.8).

**Figure 6:**
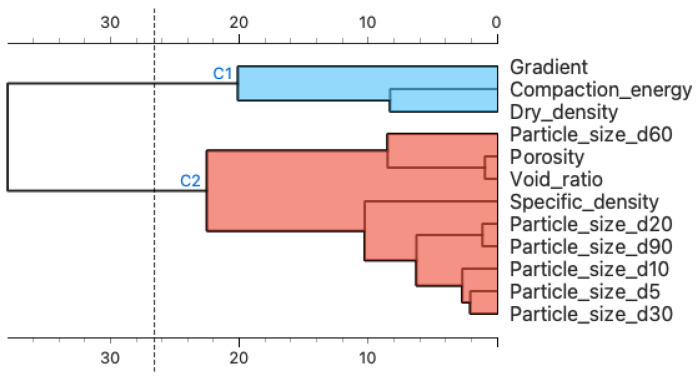
Hierarchical clustering.

In Figure 6, there is a hierarchical grouping of the properties of the tested materials based on the distance matrix between normalized data within each parameter. The analysis presents them as a dendrogram. This is a confirmation of the previously demonstrated number of clusters that occurred. It also provides insight into the distribution of the parameters across the clusters. This allows us to see how individual clusters interrelate and influence the estimate [39,49]. In Figure 6, we identify the presence of two main clusters. The parameters at the top of the hierarchical cluster analysis are gradient and particle size d60.

Table 2 shows the estimation results for the training sample. The best fit of the model estimation results to the training data was obtained for the k-NN algorithm. The coefficient of determination for this model was 0.947. For the neural network algorithm, an R^2^ result similar to that for linear regression was obtained, 0.877 and 0.829, respectively. 

For the test sample (Table 3), the results of the estimation model fit analysis for the k-NN algorithm were similar and even slightly better than for the training sample. Similarly, for linear regression whose fit coefficient was 0.844, the neural network algorithm obtained better results and an R^2^ of 0.936.

**Figure 7:**
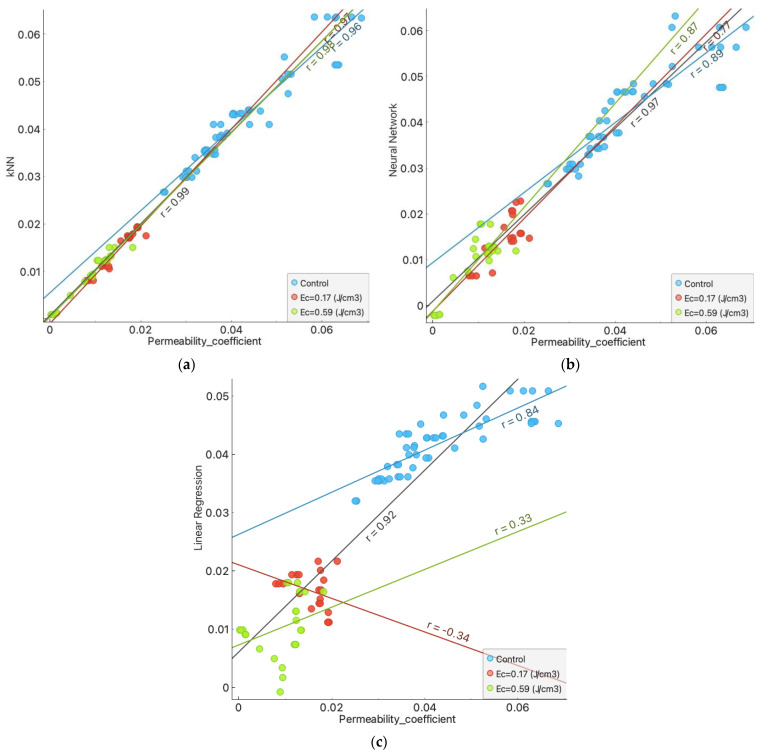
Regression plot of the prediction results of each algorithm to the test results: (**a**) k-NN algorithm. (**b**) Neural network algorithm. (**c**) Linear regression.

The estimation results versus the raw data for the different algorithms and the studied compaction energies are presented in Figure 7. In Figure 7a, the compiled estimation results for the k-NN models can be observed with a very high convergence of the estimated results for all three compaction energies. The resultant r for this comparison was 0.99. At the opposite extreme were the results for the linear regression, which did not give good predictive results in particular for the compaction energies of 0.17 and 0.59 J/cm^3^. Since the predictive results for the linear regression canceled each other out, better final R^2^ (overall) results were obtained than the data analysis based on the compaction energies allows. The partial results for this algorithm were as follows: for samples without additional compaction energy, r = 0.84; with the compaction energy of 0.17 J/cm^3^, r was −0.34; and with 0.59 J/cm^3^, r was 0.33. The resultant r was 0.92.

To explain the prediction results, SHAP was used, which is based on Shapley values. These determine the importance of each feature to the model or prediction in the same way that Shapley determines each player’s contribution to the game because Shapley theory is based on game theory. Game theory for a model can refer to a single observation of the model [50,51]. In the case of the machine learning models analyzed, which were regression models, it predicted the filtration coefficient using the material’s grain size, porosity, and density characteristics. Shapley values consider each possible feature score to determine the significance of a single feature [52]. SHAP as a method of explaining the results of the obtained machine learning models concerning civil engineering applications was also applied by Quan Tran et al., (2022) [53].

**Figure 8:**
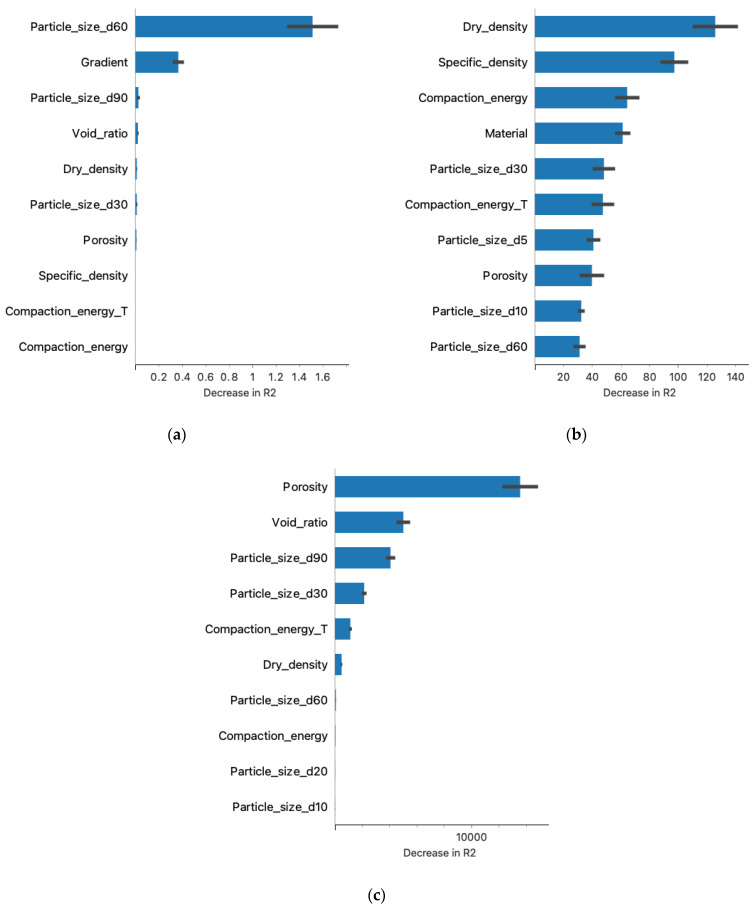
The results of the significance of the various features of the models: (**a**) k-NN. (**b**) Neural network. (**c**) Linear regression.

Figure 8 shows the results of the significance of each feature explaining the regression models, which were created based on the input data of the learned model and the reference data from the research. The analysis used these data to calculate the contribution of each feature to the model’s prediction, measuring the increase in error after the permutation of feature values for each model. The graphs indicate the 10 most significant features of each model. For the k-NN and linear regression models, the number of significant features was significantly smaller than for the neural network model. This may have affected the model’s robustness to changes in model parameters. 

If we relate this result to the earlier hierarchical analysis of the clusters (Figure 6), it is easy to see that the model considered the parameters at the top of each cluster to be the most significant and influential: gradient and particle size d60. The linear regression considered significant the parameters having the highest correlation with the filtered coefficient (Figure 5): porosity and void ratio. The situation was different with artificial neural networks, where a significant effect on R^2^ was observed for parameters both strongly and weakly correlated with the permeability coefficient, which may indicate the high robustness of the model to outliers, for example, dry density correlation of −0.9 and specific density of 0.1.

**Figure 9:**
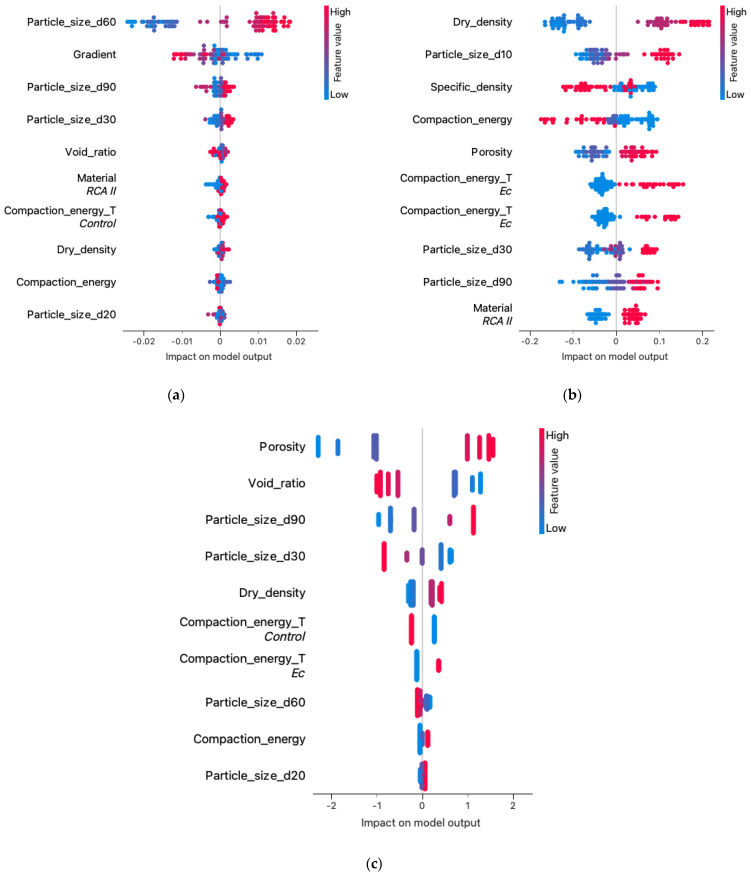
The structure feature values at the impact on the model in different algorithms: (**a**) kNN. (**b**) Neural network. (**c**) Linear regression.

Figure 9 combines the meaning of the feature with the outcomes of the feature. Each point on the graph is a Shapley value for the feature and the item instance. The position on the y-axis is determined by the feature, and the position on the x-axis is determined by the impact on the model output (SHAP value). The color represents the value of the trait from low to high. Overlapping points are separated in the y-axis direction to give the impression of a distribution of Shapley values for each feature.

The features are ordered by their importance. Figure 9 shows that higher values and importance of features have higher SHAP values, which means that higher values for features correspond to a positive class prediction in the model.

Figure 9 provides a better insight into the situation of the influence of individual data within a given material characteristic on the predictive ability of the model. Comparing the feature cluster membership results (Figure 6) and the results of Figure 9a, we observed that both focused on the significance of the influence of two material properties: particle size d60 and hydraulic gradient. The neural network algorithm (Figure 9b) featured a different parameter structure than the k-NN algorithm; the “impact on model output” values also had a larger spread. The contribution of linear regression presented in Figure 9c also shows that at least the first six features have a significant impact on model predictions. The high polarity of the impact on the model output relative to the height of the feature value may indicate the weak resistance of the model to feature changes caused by material type.

In general, SHAP provided many detailed solutions for each instance and a global interpretation of the model. This allowed for a better understanding of the characteristics of each model, its functioning, and its estimation. It also allowed us to better understand the relationships and factors influencing the studied parameter. SHAP interpreted the internal approach of machine learning models. Local explanations and influences for individual models allowed for achieving the objective of explaining the reasoning behind the prediction.

**Figure 10:**
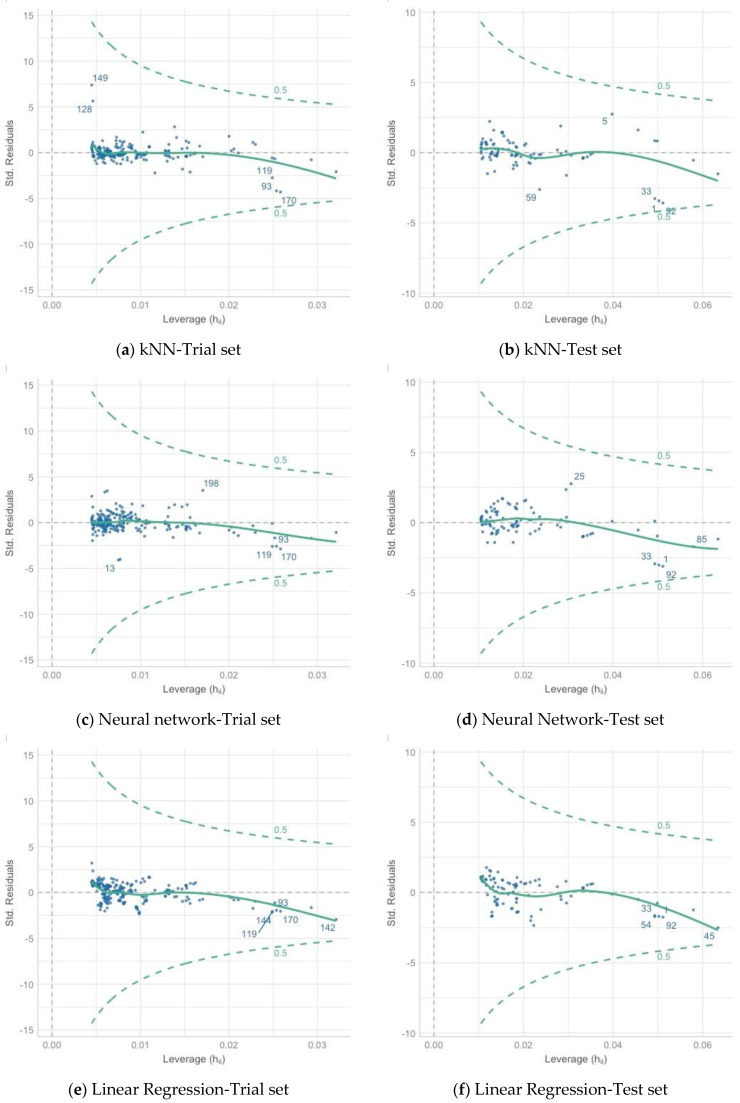
The figure of influential observations in different algorithms: k-NN: Trial set (**a**), Test set (**b**). Neural network: Trial set (**c**), Test set (**d**). Linear regression: Trial set (**e**), Test set (**f**).

The analysis of the influential variables (Figure 10) did not isolate values that had a significant impact on the models in either the training or test sets.

As a limitation of the presented study, it should be recognized that although the study used SHAP for interpretation, there are other methods for interpreting models obtained from machine learning algorithms. The study focused on the estimation of the coefficient of permeability, but the algorithms analyzed in the study as well as the methods of model interpretation may be applied to other geotechnical parameters. To predict the permeability coefficient for other anthropogenic materials, machine learning algorithms combined with explanatory methods can be helpful. Based on the analysis, extended by a larger database including other materials, it will be possible to identify influencing parameters that can be applied already at the design stage in engineering practice.

## 4. Conclusions

The main conclusions drawn from the research presented in this article are presented below:

1. The results of the study suggest that the methods of machine learning algorithms may be applicable for the prediction of the coefficient of permeability and, more broadly, geotechnical parameters.
2. To ensure the quality and reliability of the estimated model, a sufficiently large database should be provided and continuously developed. Also important is the proper preparation of the database for analysis, which is the basis for the determination of reliable models.
3. The results of the post-prediction error analysis obtained for the k-NN algorithm may indicate the correct choice of the model for estimating the coefficient of permeability for recycled anthropogenic aggregates. Error analysis for the training sample showed an RMSE error of 0.004, while the MAE was 0.002. The coefficients of determination for both the training and test sets were accordingly 0.947 and 0.980. However, taking into consideration the analysis of significant characteristics impacting the explanation of the model (Figure 8 and Figure 9), one should take into account the lower resistance of the model to changes in the characteristics of materials.
4. Given the above conclusions, the neural network model should also be considered. Admittedly, the model performs worse when analyzing errors (RMSE: 0.005–0.006 and MAE: 0.003–0.004) and R^2^ (0.877 for the trial set and 0.936 for the test set) than the model based on the k-NN algorithm, but it takes into account more features that affect the prediction of the model. As a result, it can affect lower errors when estimating the coefficient of permeability for other materials.
5. According to Darcy’s law, the dependence of gradient and filtration velocity is linear, and most of the empirical equations formed based on this relation are linear regression. The research presented in this article proves that this model is not suitable for generalizing predictions based on the features and parameters of anthropogenic materials and allowing at the same time the consideration that machine learning algorithms are better suited to these prediction tasks.
6. From the perspective of the k-NN algorithm, it is necessary to recognize the importance of the clustered hierarchical analysis (Figure 6) since the parameters occupying the leading positions here have a significant impact (Figure 8) on the formation of the model.
7. The analysis should be repeated for other anthropogenic and post-industrial materials used in civil engineering to validate the usefulness of the analyzed algorithms.
8. The use of interpretive methods such as SHAP allows for better insight into the performance of the model and provides valuable information about parameters that have an important impact on the final model and are a significant part of the study. We suggest using interpretive machine learning methods to support decision criteria in civil engineering applications.

## Figures and Tables

**Table 1:** Average values of parameters of physical properties of materials.

Description	Mean Value
Particle size d5 [mm]	0.27
Particle size d10 [mm]	0.60
Particle size d20 [mm]	1.78
Particle size d30 [mm]	3.38
Particle size d60 [mm]	11.06
Particle size d90 [mm]	23.38
Dry density [g/cm^3^]	1.35
Porosity [-]	0.465
Void ratio [-]	0.902

**Table 2:** Performance results of using the various algorithms to predict the permeability coefficient.

Trial Set				
Model	MSE	RMSE	MAE	R^2^
kNN	0.000	0.004	0.002	0.947
Neural network	0.000	0.006	0.004	0.877
Linear regresion	0.000	0.007	0.005	0.829

**Table 3:** Performance results of using the various algorithms to predict the permeability coefficient.

Test Set				
Model	MSE	RMSE	MAE	R^2^
kNN	0.000	0.003	0.002	0.980
Neural network	0.000	0.005	0.003	0.936
Linear regresion	0.000	0.007	0.006	0.844

## Data Availability

Not applicable.
